# Toilet behaviors and lifestyle factors in anorectal diseases: a cross-sectional analysis

**DOI:** 10.3389/fsurg.2025.1683286

**Published:** 2025-11-18

**Authors:** Cenk Yazkan, Samet Şahin, Burak Yavuz, Anar Mammadov, Önder Özcan, Özcan Dere

**Affiliations:** 1 Department of General Surgery, Faculty of Medicine, Muğla Sıtkı Koçman University, Muğla, Türkiye; 2 Department of General Surgery, Kozan State Hospital, Adana, Türkiye; 3 Faculty of Medicine, Muğla Sıtkı Koçman University, Muğla, Türkiye

**Keywords:** anorectal diseases, defecation, constipation, hemorrhoids, proctology

## Abstract

**Background:**

Anorectal diseases including hemorrhoids, anal fissures, and fistulas significantly impact quality of life, yet the relationship between toilet behaviors and disease development remains poorly understood.

**Methods:**

This cross-sectional study conducted at Muğla Sıtkı Koçman University compared 211 proctology patients with anorectal diseases to 369 healthy controls. Data on demographics, toilet behaviors, hygiene practices, and lifestyle factors were collected through structured interviews.

**Results:**

Among 580 participants (211 patients vs. 369 controls), patients were significantly older (46.0 ± 14.0 vs. 42.2 ± 16.4 years; *p* = 0.002) and predominantly male (76.3% vs. 45.0%; *p* < 0.001). They also had higher rates of constipation (>1/week: 18.9% vs. 5.7%; *p* < 0.001), back-to-front wiping (59.2% vs. 45.5%; *p* = 0.002), and prolonged toilet sitting (>20 min: 8.1% vs. 0.5%; *p* < 0.001), whereas smartphone use during defecation was lower (26.1% vs. 37.4%; *p* = 0.001).

**Conclusion:**

This study demonstrates significant associations between specific toilet behaviors and anorectal disease development. The findings suggest that constipation frequency, wiping patterns, and prolonged toilet sitting represent modifiable risk factors, while the inverse relationship with smartphone use warrants further investigation. These results support targeted behavioral interventions in anorectal disease prevention.

## Highlights

What is already known:

• Prolonged toilet sitting and straining are implicated in anorectal disease, but behavioural evidence has been inconsistent.
• Fibre and hydration reduce stool hardness and defecation time.

What this study adds:

• Compared with controls, patients showed higher weekly constipation (18.9% vs. 5.7%), more sessions >20 min (8.1% vs. 0.5%), and more back-to-front wiping (59.2% vs. 45.5%).
• Smartphone use was lower in patients (26.1% vs. 37.4%), suggesting reverse causation (behaviour changes after symptom onset).

How this might affect practice/policy:

• Clinicians should screen and counsel on constipation, limit toilet time to ≤3–5 min, and reinforce front-to-back wiping.
• Programmes and patient leaflets can target these low-cost, actionable behaviours to reduce symptom burden.

## Introduction

Anorectal diseases, encompassing hemorrhoids, anal fissures, and anal fistulas, represent a significant global health burden affecting millions worldwide. Recent epidemiological studies demonstrate hemorrhoid prevalence ranging from 4.4% to 38.9% in adult populations, with peak incidence occurring between ages 45–65 years (1). Anal fissures show an annual incidence of 0.11% with lifetime risk reaching 7.8% (2), while anal fistulas affect approximately 18.37 per 100,000 individuals globally (3). Dietary modification remains a cornerstone of anorectal disease prevention. In particular, adequate fiber intake and hydration reduce stool hardness, shorten defecation time, and lower straining, thereby mitigating venous congestion and mucosal trauma Contemporary guidelines generally recommend ∼25–38 g/day of fiber with sufficient daily fluid to maintain soft, formed stools (4). In populations with prevalent constipation, structured fiber supplementation and fluid targets have been associated with fewer symptomatic hemorrhoids and fissure episodes and with improved toileting efficiency (5). These behavioral levers are low-cost, scalable, and readily integrated into routine counseling, making them especially relevant to proctology practice.

Despite their prevalence, the relationship between daily toilet behaviors and anorectal disease development remains incompletely understood. Current pathophysiological models emphasize increased intra-abdominal pressure, venous congestion, and tissue trauma as key mechanisms, yet specific behavioral risk factors have not been systematically characterized (6). Recent research suggests smartphone use during defecation increases hemorrhoid risk by 46% (7), while prolonged toilet sitting creates extreme pressure on anal tissues through gravity-mediated blood pooling (8).

Cultural and behavioral factors significantly influence toilet practices across populations. Traditional squatting positions may reduce straining and defecation time compared to Western sitting toilets (9), while hygiene practices including wiping patterns show considerable variation. The front-to-back wiping technique is universally recommended to prevent bacterial transfer, yet adherence varies significantly (10).

Constipation represents a well-established risk factor, with recent studies demonstrating adjusted odds ratios of 4.32 for hemorrhoid development among constipated individuals (11). However, the specific frequency thresholds and interaction with toilet behaviors remain unclear. Understanding these relationships is crucial for developing evidence-based prevention strategies.

This cross-sectional study aimed to comprehensively evaluate toilet behaviors, hygiene practices, and lifestyle factors among patients with anorectal diseases compared to healthy controls. We hypothesized that specific modifiable behaviors would demonstrate significant associations with disease development, providing targets for clinical intervention.

## Methods

This cross-sectional study was conducted at the Department of General Surgery, Muğla Sıtkı Koçman University Training and Research Hospital, Turkey, between December 2023 and June 2025. The protocol was approved by the institutional ethics committee (Protocol No. 230104 Date: Nov 23th 2023), and all participants provided written informed consent.

Consecutive adult patients (aged 18–80 years) presenting to the proctology outpatient clinic with a confirmed diagnosis of hemorrhoids, anal fissures, or anal fistulas were enrolled (*n* = 211). Exclusion criteria included inflammatory bowel disease, colorectal malignancy, prior anorectal surgery, pregnancy, and inability to complete the questionnaire. A control group of healthy volunteers (*n* = 369) was recruited after screening to exclude any history of anorectal symptoms, prior gastrointestinal surgery, or current digestive disorders. Written informed consent was obtained from all participants prior to enrollment.

Data were collected via structured, face-to-face interviews conducted by trained research staff. A standardized symptom assessment questionnaire was administered to all proctology patients, recording presence of pain, bleeding, discharge, and pruritus regardless of primary diagnosis. Demographic and clinical information comprised age, sex, body mass index, blood type, comorbidity status, disease subtype, presenting symptoms (pain, bleeding, discharge, pruritus), smoking, and alcohol use. Lifestyle and dietary factors included daily standing time, frequency of physical activity, breakfast habits, overall meal and fast-food consumption, bread intake, and daily fluid consumption. Toilet behaviors and hygiene practices captured defecation frequency, constipation episodes, toilet type, duration of toilet use, use of smartphones or reading materials during defecation, wiping technique (front-to-back vs. back-to-front) and water cleansing routines. Data completeness was excellent, with missing values accounting for less than 1% across all variables. Listwise deletion was employed for statistical analyses, with cases containing substantial missing data excluded from models to ensure validity of results.

Statistical analyses were performed using SPSS version 28.0 (IBM Corp., Armonk, NY). Continuous variables were tested for normality by the Kolmogorov–Smirnov test and reported as mean ± standard deviation or median (interquartile range) as appropriate; categorical variables were expressed as counts and percentages. Between-group comparisons employed independent *t*-tests or Mann–Whitney *U* tests for continuous data and chi-square tests for categorical data. A *p*-value of less than 0.05 was considered statistically significant. A *post-hoc* power analysis was conducted using G*Power 3.1 showed that the sample size of 211 patients and 369 controls provided 99% statistical power to detect the observed difference in constipation frequency (18.9% vs. 5.7%, *p* < 0.001) at *α* = 0.05 level.

## Results

A total of 580 participants were enrolled, including 211 patients with anorectal diseases (hemorrhoids, fissures, or fistulas) and 369 healthy controls (Table 1). Patients were significantly older than controls (46.0 ± 14.0 vs. 42.2 ± 16.4 years; *p* = 0.002) and predominantly male (76.3% vs. 45.0%; *p* < 0.001). Body mass index did not differ between groups (27.5 ± 4.5 vs. 26.9 ± 5.1; *p* = 0.071). Blood group O (Rh− and Rh+) was more frequent among patients than controls (50.3% vs. 42.5%; *p* = 0.013). Comorbidity rates were comparable (41.6% vs. 43.2%; *p* = 0.707). Among patients, diagnoses were distributed as follows: hemorrhoids 41.7%, anal fissures 38.4%, and anal fistulas 19.9%. The most common presenting symptom was pain (53.6%), followed by bleeding and discharge (each 33.2%), and pruritus (21.8%). Smoking (43.6% vs. 39.2%; *p* = 0.304) and alcohol use (33.7% vs. 30.0%; *p* = 0.360) did not differ significantly (Table 1).

**Table 1:** Demographical data.

Parameter	Patient (*n* = 211)	Control (*N* = 369)	*p* value
Age	46.0 ± 14.0	42.2 ± 16.4	**0.002**
Gender			
Male	76.3% (161)	45.0% (166)	**<0.001**
Female	23.7% (50)	55.0% (203)	
BMI	27.5 ± 4.5	26.9 ± 5.1	0.071
Blood type			
O			**0** **.** **013**
Rh−	6.2% (13)	2.7% (10)	
Rh+	44.1% (93)	39.8% (147)	
A			
Rh−	5.2% (11)	8.1% (30)	
Rh+	26.1% (55)	31.4% (116)	
B			
Rh−	2.4% (5)	0	
Rh+	11.8% (25)	13.6% (50)	
AB			
Rh−	0	0.5% (2)	
Rh+	4.3% (9)	3.8% (14)	
Comorbidities			
Yes	41.6% (84)	43.2% (144)	0.707
No	58.4% (118)	56.8% (189)	
Diagnosis			
Hemmorhoids	41.7% (88)	-	-
Fistula	19.9% (42)	-	
Fissure	38.4% (81)	-	
Symptoms			
Pain	53.6% (113)	-	-
Pruritus	21.8% (46)	-	
Bleeding	33.2% (70)	-	
Discharge	33.2% (70)	-	
Smoking			
Yes	43.6% (92)	39.2% (144)	0.304
No	56.4% (119)	60.8% (223)	
Alcohol			
Yes	33.7% (70)	30.0% (110)	0.360
No	66.3% (138)	70.0% (257)	

Statistically significant results (*p* < 0.05) are indicated in bold.

Standing time differed markedly between groups (Table 2): fewer patients stood >6 h per day (*p* < 0.001), with higher proportions standing <2 h (13.5% vs. 7.1%) and 2–4 h (23.2% vs. 14.2%). Sedentary behavior was more common among patients (21.4% vs. 15.5%; *p* = 0.021). Breakfast habits and fast-food consumption were similar (*p* = 0.312 and *p* = 0.434), whereas daily bread intake was marginally higher in patients (median 125 g [IQR 50–250] vs. 125 g [IQR 50–250]; *p* = 0.030). Fluid intake and overall meal frequency did not differ.

**Table 2:** Dietary and exercise habits

Parameter	Patient (*n* = 211)	Control (*N* = 369)	*p* value
Standing duration			
<2 h	13.5% (28)	7.1% (26)	**<0** **.** **001**
2–4 h	23.2% (48)*	14.2% (52)	
4–6 h	11.1% (23)	16.3% (60)	
6–8 h	17.9% (37)*	29.4% (108)	
8–10 h	18.8% (39)	17.2% (63)	
>10 h	15.5% (32)	15.8% (58)	
Exercise			
Sedentary	21.4% (44)	15.5% (56)	**0** **.** **021**
Low	35.9% (74)*	32.1% (116)	
Moderate	35.0% (72)	32.1% (116)	
High	7.8% (16)	4.4% (16)	
Routine breakfast			
No	18.7% (39)	22.2% (82)	0.312
Yes	81.3% (170)	77.8% (287)	
Daily no of meals			
1	2.9% (6)	1.6% (6)	0.07
2	36.8% (77)	46.9% (172)	
3	56.0% (117)	46.0% (169)	
>3	4.3% (9)	5.4% (20)	
Monthly fast-food intake	2.00 (0.00–5.00)	2.00 (1.00–5.00)	0.434
Daily fluid intake	2.00 (1.50–3.00)	2.00 (1.50–2.50)	0.175
Daily bread intake (grams)	125.00 (50.00–250.00)	125.00 (50.00–250.00)	**0** **.** **03**

Statistically significant results (*p* < 0.05) are indicated in bold.

* *p* < 0.05 compared to control group.

There were no significant differences in toilet type (*p* = 0.087) or diarrhea frequency (*p* = 0.280) (Table 3). However, constipation ≥1 episode/week was more prevalent in patients (18.9% vs. 5.7%; *p* < 0.001), with fewer patients reporting <1 episode/month (49.0% vs. 63.6%; *p* < 0.001). Wiping direction differed: back-to-front wiping was reported by 59.2% of patients vs. 45.5% of controls (*p* = 0.002). Prolonged toilet durations (>20 min) were more frequent in patients (8.1% vs. 0.5%; *p* < 0.001). Use of smartphones during defecation was less common among patients (26.1% vs. 37.4%; *p* = 0.001).

**Table 3:** Toilet habits

Parameter	Patient (*n* = 211)	Control (*N* = 369)	*p* value
Toilet type			
Sitting	74.4% (157)	77.5% (286)	0.087
Squat	20.9% (44)	20.9% (77)	
Both	4.7% (10)	1.6% (6)	
Number of diarrhea			
<1/month	75.1% (154)	76.4% (265)	0.280
1–2/month	18.0% (37)	18.4% (64)	
1/week	2.4% (5)	3.5% (12)	
>1/week	4.4% (9)	1.7% (6)	
Number of constipation			
<1/month	49.0% (101)*	63.6% (222)	**<0** **.** **001**
1–2/month	20.4% (42)	18.3% (64)	
1/week	11.7% (24)	12.3% (43)	
>1/week	18.9% (39)*	5.7% (20)	
Wet wipe usage			
Yes	19.9% (42)	26.6% (98)	0.072
No	80.1% (169)	73.4% (271)	
Water usage after toilet			
Yes	98.6% (208)	96.2% (355)	0.103
No	1.4% (3)	3.8% (14)	
Cleaning direction			
Back to front	59.2% (125)*	45.5% (168)	**0** **.** **002**
Front to back	38.4% (81)*	53.4% (197)	
Both	2.4% (5)	1.1% (4)	
Drying			
Yes	92.9% (196)	92.7% (342)	0.926
No	7.1% (15)	7.3% (27)	
Weekly defecation number			
<3	13.3% (28)	10.8% (40)	0.059
4–7	51.7% (109)	62.9% (232)	
8–14	20.4% (43)	15.2% (56)	
14+	10.9% (23)	6.5% (24)	
Mean defecation duration			
<5 min	35.5% (75)	42.3% (156)	**<0** **.** **001**
5–10 min	32.2% (68)	31.4% (116)	
10–15 min	16.6% (35)	14.9% (55)	
15–20 min	6.2% (13)	6.5% (24)	
>20 min	8.1% (18)*	0.5% (2)	
Telephone/reading habits in toilet			
No	72% (152)*	60.4% (223)	**0** **.** **001**
Published	3% (1.4)*	0	
Phone	26.1% (55)*	37.4% (138)	
Combined	0.5% (1)	2.2% (8)	

Statistically significant results (*p* < 0.05) are indicated in bold.

* *p* < 0.05 compared to control group.

Significant differences in lifestyle and toileting habits emerged across patient subgroups (Table 4). Constipation ≥1 episode/week was most common in hemorrhoid patients (24.7%), followed by fissure (19.5%) and fistula (12.5%) groups, compared with 5.7% of controls (*p* < 0.001). Back-to-front wiping occurred in 60.2% of hemorrhoid, 52.4% of fissure, and 61.7% of fistula patients vs. 45.5% of controls (*p* = 0.032). Sedentary behavior was highest among fissure patients (32.5%) compared to 15.5% of controls (*p* = 0.017). Toilet sessions lasting <5 min were less frequent in the hemorrhoid group (25.0% vs. 42.3% in controls), whereas sessions >20 min were markedly more frequent in hemorrhoid (12.5%), fissure (4.8%), and fistula (4.9%) patients than in controls (0.5%) (*p* < 0.001). Finally, abstaining from any smartphone or reading-material use during defecation was reported by 72.7% of hemorrhoid, 69.0% of fissure, and 72.8% of fistula patients vs. 60.4% of controls (*p* = 0.017).

**Table 4:** Comparison of lifestyle and habits among groups

Parameter	Hemmorhoids	Fissure	Fistula	Control	*p* value
Number of constipation					
<1/month	37.6% (32)	43.9% (18)	63.7% (51)	63.6% (222)	**<0** **.** **001**
1–2/month	27.1% (23)	17.1% (7)	15.0% (12)	18.3% (64)	
1/week	10.6% (9)	19.5% (8)	8.8% (7)	12.3% (43)	
>1/week	24.7% (21)	19.5% (8)	12.5% (10)	5.7% (20)	
Cleaning direction					
Back to front	60.2% (53)	52.4% (22)	61.7% (50)	45.5% (168)	**0** **.** **032**
Front to back	37.5% (33)*	45.2% (19)	35.8% (29)*	53.4% (197)	
Both	2.3% (2)	2.4% (1)	2.5% (2)	1.1% (4)	
Exercise					
Sedentary	18.8% (16)	32.5% (13)*	18.5% (15)	15.5% (56)	**0** **.** **017**
Low	40% (34)	35% (14)	32.1% (26)	47.9% (173)	
Moderate	32.9% (28)	32.5% (13)	38.3% (31)	32.1% (116)	
High	8.2% (7)	0	11.1% (9)	4.4% (16)	
Mean defecation duration					
<5 min	25% (22)*	42.9% (18)	43.2% (35)	42.3% (156)	**<0** **.** **001**
5–10 min	23.9% (21)	38.1% (16)	38.3% (31)	31.4% (116)	
10–15 min	28.4% (25)	7.1% (3)	8.6% (7)	14.9% (55)	
15–20 min	8% (7)	4.8% (2)	4.9% (4)	6.5% (24)	
>20 min	12.5% (11)*	4.8% (2)*	4.9% (4)*	0.5% (2)	
Telephone/reading habits in toilet					
No	72.7% (64)	69.0% (29)	72.8% (59)	60.4% (223)	**0** **.** **017**
Published	1.1% (1)	0	2.5% (2)*	0	
Phone	25.0% (22)	31.0% (13)	24.7% (20)	37.4% (138)	
Combined	1.1% (1)	0	0	2.2% (8)	

Statistically significant results (*p* < 0.05) are indicated in bold.

* *p* < 0.05 compared to control group.

## Discussion

This comprehensive cross-sectional analysis provides novel insights into the relationship between toilet behaviors and anorectal disease development. Our findings demonstrate significant associations with multiple modifiable risk factors, offering potential targets for prevention strategies.

The observed age distribution (mean 46.0 years) and male predominance (76.3%) in our patient cohort align closely with recent international epidemiological data. Studies report peak hemorrhoid incidence at ages 45–65 years with 66.7% male predominance (11), while some analyses consistently demonstrate 2:1 male-to-female ratios for anal fistulas (3). This demographic consistency suggests our findings may be generalizable across diverse populations.

The striking male predominance in our study (76.3% vs. 45.0% controls) exceeds most published reports, possibly reflecting cultural factors in healthcare-seeking behavior or occupational risk factors prevalent in our Turkish population. Recent studies suggest men may experience more severe symptoms requiring medical attention, while women more frequently manage symptoms conservatively (12).

Our finding of 18.9% vs. 5.7% weekly constipation frequency strongly supports established pathophysiological mechanisms. Recent cohort studies demonstrate adjusted odds ratios of 4.32 for hemorrhoid development among constipated individuals (11). This association operates through increased intra-abdominal pressure during straining, creating coordinated pressure changes from 18 ± 4 cmH_2_O to 68 ± 15 cmH_2_O during attempted evacuation (13).

The mechanistic pathway involves dyssynergic defecation patterns characterized by paradoxical puborectalis and external anal sphincter contraction, preventing establishment of the negative anorectal gradient necessary for smooth evacuation (14). Chronic increased intra-abdominal pressure leads to venous congestion and progressive tissue damage, ultimately manifesting as hemorrhoidal disease.

Current American Gastroenterological Association guidelines emphasize polyethylene glycol and fiber supplementation as first-line interventions (15), supporting early aggressive management of constipation to prevent anorectal complications. The dramatic 16-fold increase in prolonged toilet sitting (>20 min: 8.1% vs. 0.5%) represents one of our most striking findings. Recent Italian studies demonstrate linear associations between toilet sitting time and hemorrhoid severity (8), while clinical guidelines recommend maximum 3–5 min sessions to prevent tissue damage.

The pathophysiological mechanism involves gravity-mediated blood pooling in dependent hemorrhoidal vessels, creating extreme pressure on rectal and anal tissues. This process is exacerbated by the anatomical toilet position, which creates a dependent loop in hemorrhoidal circulation. The TONE mnemonic (3 min, Once daily, No straining, Enough fiber) has emerged as an evidence-based framework for optimal defecation habits (16).

The unexpected inverse relationship between smartphone use and anorectal disease (26.1% vs. 37.4%, *p* = 0.001) contradicts recent controlled studies demonstrating 46% increased hemorrhoid risk among smartphone users (7). This paradox likely reflects reverse causation, where patients with symptomatic anorectal disease consciously modify their toilet behaviors to minimize discomfort and expedite defecation.

Recent Turkish studies demonstrate that 64.7% of hemorrhoid patients vs. 38.4% of controls typically use smartphones, but symptomatic patients often abandon this practice due to urgency and discomfort (17). This behavioral adaptation may explain our counterintuitive findings and highlights the importance of longitudinal rather than cross-sectional study designs for behavioral risk factor assessment.

The prevalence of back-to-front wiping among patients (59.2% vs. 45.5%, *p* = 0.002) directly contradicts evidence-based clinical recommendations. Front-to-back wiping is universally recommended to prevent bacterial transfer from anus to urethra, particularly crucial for women to prevent urinary tract infections (10). The reverse technique may contribute to perianal irritation and bacterial contamination, potentially perpetuating inflammatory processes in existing anorectal disease.

Notably, overall dietary patterns and hydration were broadly similar between patients and controls in this cohort (e.g., meal frequency and daily fluid intake did not differ meaningfully), suggesting that disease associations were driven less by global nutrition profiles and more by specific behaviors such as constipation frequency, wiping direction, and prolonged toilet sitting. This nuance underscores the importance of targeted habit counseling rather than generic dietary advice.

Disease-specific patterns in our subgroup analysis further refine these inferences. Hemorrhoid patients exhibited the highest rates of both weekly constipation and prolonged defecation (>20 min), consistent with pathophysiologic models linking straining and time-dependent venous engorgement to symptomatic disease. Fissure patients showed the greatest sedentary behavior, aligning with clinical observations that inactivity aggravates evacuation difficulty and sphincter spasm. In fistula and hemorrhoid groups, back-to-front wiping was more common than in controls, plausibly contributing to perianal irritation and contamination that may perpetuate symptoms. Taken together, these contrasts argue for tailored counseling: constipation reduction and strict time-limits on the toilet for hemorrhoids (18) and activity promotion and stool-softening strategies for fissures (19).

Our findings support a multimodal prevention approach targeting identified behavioral risk factors. Primary interventions should focus on constipation prevention through evidence-based dietary modifications (25–38 g daily fiber intake), adequate hydration, and regular physical activity. The 2023 AGA/ACG guidelines provide robust evidence for polyethylene glycol, fiber supplementation, and lifestyle modifications (15).

Secondary prevention strategies should address toilet behaviors through patient education emphasizing proper technique: front-to-back wiping, limited toilet time (<5 min), avoidance of straining, and appropriate response to natural urges. The integration of behavioral counseling into routine proctology care may significantly impact disease prevention and progression.

Tertiary prevention for symptomatic patients should include comprehensive lifestyle modification programs combining dietary counseling, exercise prescription, and toilet habit education. Recent studies demonstrate that 77% of chronic constipation patients benefit from structured fiber supplementation programs with sustained improvement (20).

Importantly, our cross-sectional design captures current behaviors rather than pre-disease habits. The inverse relationship between smartphone use and toilet duration in our patient group may reflect behavioral modifications adopted after symptom onset. Patients experiencing pain or urgency may consciously avoid distractions to expedite defecation. This reverse causation hypothesis can only be tested through prospective cohort studies tracking behavioral changes from pre-disease states through symptom development. The inability to determine temporal relationships between behaviors and disease onset represents a fundamental limitation of our cross-sectional methodology.

This study highlights immediately actionable behaviours for routine proctology care. Compared with controls, patients reported higher weekly constipation (18.9% vs. 5.7%), more prolonged toilet sitting (>20 min: 8.1% vs. 0.5%), and more back-to-front wiping (59.2% vs. 45.5%), indicating clear counselling targets. Clinicians should systematically screen for constipation, toilet time, and wiping direction, and provide brief education to limit each session to ≤3–5 min, avoid straining, adopt front-to-back wiping, and maintain adequate fibre intake (∼25–38 g/day) with hydration and regular activity. The lower smartphone use among patients likely reflects post-symptom adaptation (reverse causation) and should not divert counselling away from time-on-toilet. Embedding a simple checklist into EMR or discharge materials can standardise assessment and follow-up.

Future research should employ prospective longitudinal validated designs to establish temporal relationships between behaviors and disease development. Randomized controlled trials of behavioral interventions are needed to determine the efficacy of targeted toilet habit modifications. Smartphone intervention studies specifically addressing usage cessation during defecation may clarify the apparent protective association observed.

## Limitations

Several limitations must be acknowledged. This hospital-based cross-sectional design precludes causal inference and may over-represent symptomatic cases. Toilet behaviours (e.g., sitting time, smartphone use) were self-reported and not objectively timed, introducing recall and social-desirability bias. Behaviours were recorded at presentation and may reflect post-diagnosis adaptations (reverse causality), particularly relevant to the smartphone–duration findings and pain/urgency. Haemorrhoid severity was not systematically graded using the Goligher classification, and acute thrombosed haemorrhoids were not captured; thus, the diagnosis-symptom (pain vs. bleeding) mismatch cannot be fully resolved. The questionnaire used in this study was developed considering regional and cultural factors specific to the Turkish population. While formal validation procedures were not conducted, the significant associations observed between behavioral factors and clinical outcomes suggest adequate construct validity. Prospective, multi-centre studies with standardized disease grading and objective digital measures are needed.

## Conclusion

Our analysis reveals that constipation frequency, prolonged toilet sitting, and inefficient wiping patterns are significantly associated with anorectal disease, highlighting clear, modifiable targets for intervention, while the unexpected inverse link with smartphone use merits further study; consistent with global epidemiological trends, these findings support integrating behavioral counseling and routine assessment of toilet habits into proctological care for primary prevention, with longitudinal research needed to confirm causality and evaluate intervention effectiveness.

## Data Availability

The raw data supporting the conclusions of this article will be made available by the authors, without undue reservation.
